# Supplementary material for: Evaluation of Choline Metabolic Genes in the Liver of the Dam as Candidates for Mediating Choline’s Efficacy in Mitigating Ethanol-Induced Cell Death in the Neural Tube: A Preliminary Analysis
Source: Genes (Basel). 2025 Dec 31;17(1):42. doi: 10.3390/genes17010042 (PMC12841201; doi:10.3390/genes17010042)
Supplement: Supplementary file 1 [file genes-17-00042-s001.zip › genes-4012834-supplementary.pdf]

Please find the following supplementary material available below:

Liver Proteome Datasets:

Aldh7a1: https://genenetwork.org/show_trait?trait_id=Q9DBF1_SAYSQIR_2&dataset=EPFL-ETHZ-BXD-LivProtAvg-CD-1119

Bhmt:
https://genenetwork.org/show_trait?trait_id=O35490_AIAEELAPER_2&dataset=EPFL-ETHZ-BXD-LivProtAvg-CD-1119

Bhmt2:
https://genenetwork.org/show_trait?trait_id=Q91WS4_AGLWTPEAVVEHPSAVR_3&dataset=EPFL-ETHZ-BXD-LivProtAvg-CD-1119

Cept1:
https://genenetwork.org/show_trait?trait_id=Q8BGS7_LFQLPTPPLSR_2&dataset=EPFL-ETHZ-BXD-LivProtAvg-CD-1119

Slc44a1:
https://genenetwork.org/show_trait?trait_id=A2AMH4_LPVPASAPIPFFHR_3&dataset=EPFL-ETHZ-BXD-LivProtAvg-CD-1119

Link to analyses with percent cell death data and sample sizes:

https://liveutk-my.sharepoint.com/:x:/g/personal/tchowdh2_uthsc_edu/IQC99A-ZWm6eQZmKjlt3LOfWAac0LHTf_C7Kt0PgoxtemB4?e=c2PD2q
